# The association between ambient temperature and antimicrobial resistance of *Klebsiella pneumoniae* in China: a difference-in-differences analysis

**DOI:** 10.3389/fpubh.2023.1158762

**Published:** 2023-06-08

**Authors:** Yingchao Zeng, Weibin Li, Manzhi Zhao, Jia Li, Xu Liu, Lin Shi, Xinyi Yang, Haohai Xia, Shifang Yang, Lianping Yang

**Affiliations:** ^1^School of Public Health, Sun Yat-sen University, Guangzhou, China; ^2^Department of Pulmonary and Critical Care Medicine, Guangdong Provincial People’s Hospital (Guangdong Academy of Medical Sciences), Southern Medical University. Guangzhou, Guangdong, China; ^3^Department of Pharmacy, The First Affiliated Hospital, Sun Yat-sen University, Guangzhou, China; ^4^Department of Infectious Disease, The Fifth Affiliated Hospital, Sun Yat-sen University, Zhuhai, China

**Keywords:** antimicrobial resistance, ambient temperature, *Klebsiella pneumoniae*, socioeconomic status, difference-in-differences

## Abstract

**Introduction:**

Antimicrobial resistance (AMR) of *Klebsiella pneumoniae* (*K. pneumoniae*) poses a significant global public health threat and is responsible for a high prevalence of infections and mortality. However, knowledge about how ambient temperature influences the AMR of *K. pneumoniae* is limited in the context of global warming.

**Methods:**

AMR data of 31 Chinese provinces was collected from the China Antimicrobial Resistance Surveillance System (CARSS) between 2014 and 2020. Socioeconomic and meteorological data were collected from the China Statistical Yearbook during the same period. A modified difference-in-differences (DID) approach was applied to estimate the association between ambient temperature and third-generation cephalosporin-resistant *K. pneumoniae* (3GCRKP) and carbapenem-resistant *K. pneumoniae* (CRKP). Furthermore, moderating effects of socioeconomic factors were also evaluated.

**Results:**

Every 1°C increase in annual average temperature was associated with a 4.7% (relative risk (RR):1.047, 95% confidence intervals (CI): 1.031–1.082) increase in the detection rate of 3GCRKP, and a 10.7% (RR:1.107, 95% CI: 1.011–1.211) increase in the detection rate of CRKP. The relationships between ambient temperature and 3GCRKP and CRKP were found to be moderated by socioeconomic status (GDP *per capita*, income *per capita*, and consumption *per capita*; the interaction *p*-values <0.05), where higher economic status was found to strengthen the effects of temperature on the detection rate of 3GCRKP and weaken the effects on the detection rate of CRKP.

**Discussion:**

Ambient temperature was found to be positively associated with AMR of *K. pneumoniae*, and this association was moderated by socioeconomic status. Policymakers should consider the impact of global warming and high temperatures on the spread of 3GCRKP and CRKP when developing strategies for the containment of AMR.

## 1. Introduction

Antimicrobial resistance (AMR) is a serious global issue of increasing concern (1), with *Klebsiella pneumoniae* (*K. pneumoniae*) identified as a global priority AMR pathogen by the World Health Organization (WHO). *K. pneumoniae* imposes a significant burden on healthcare systems (2), particularly in low- and middle-income countries (3–5). In China, a multi-center investigation involving 25 tertiary hospitals across 14 Chinese provinces found that *K. pneumoniae* accounted for 73.9% of carbapenem-resistant *Enterobacterales* (CRE) infections, making it one of the most prevalent AMR pathogens (6). The high prevalence of antimicrobial resistant *K. pneumoniae* can be attributed to both endogenous and exogenous factors (7). *K. pneumoniae* has a wide ecological distribution, diverse DNA composition, high plasmid load, and great AMR gene diversity, which make AMR genes of *K. pneumoniae* more easily disseminated from environmental microbes to clinically important pathogens (8). Moreover, environmental factors, such as seasonal change and temperature fluctuations (9–12), may also contribute to the dissemination of *K. pneumoniae* (13–15).

*Klebsiella pneumoniae* is a common cause of severe Gram-negative bacteria infections in people (6), and these infections exhibit seasonal peaks that more frequently occur in the summer (16–18). However, limited empirical evidence exists on the association between ambient temperature and the antimicrobial resistance, let alone the *K. pneumoniae* (19). A previous global study indicated a positive correlation between temperature and the aggregate AMR index, which included *K. pneumoniae* resistant to third-generation cephalosporins and carbapenems, but did not separate the independent effect of temperature in the multivariable model or assess the impact of temporal trends on AMR (20). Similarly, an ecological study conducted in the United States (21) and a 17-year multi-country study conducted in European areas also identified a positive association between temperature and the AMR of *K. pneumoniae* (22). However, neither of the above studies considered the impact of temporal trends or other potential factors associated with AMR, such as economic status and healthcare resource factors (20). Ambient temperature likely has cumulative impacts on AMR (15). Evaluating the hysteresis impact of ambient temperature on AMR has been a matter of great account under climate change.

As global warming exacerbates, China is currently experiencing its most prolonged and severe heat wave on record (23, 24). It is imperative to investigate how rising temperatures may impact the AMR of *K. pneumoniae*. In this study, we aimed to estimate the association between temperature and the prevalence of third-generation cephalosporin-resistant *K. pneumoniae* (3GCRKP) or carbapenem-resistant *K. pneumoniae* (CRKP) across 31 provinces in China, using an extended difference-in-differences (DID) approach, in order to provide policymakers with valuable information and critical insights on strategies to control AMR.

## 2. Materials and methods

### 2.1. Data collection

The China Antimicrobial Resistance Surveillance System (CARSS, http://www.carss.cn/) was used as the primary microbiology data source for this analysis. CARSS is a nationally representative database that reports AMR rates by province. It covers 1,371 hospitals, including 1,019 tertiary hospitals and 352 secondary hospitals, across 31 provinces, autonomous regions, and municipalities directly under the Central Government. These areas are considered provinces in this study as they share the same administrative level. Antimicrobial susceptibility tests were conducted in accordance with the guidelines and criteria for quality control set forth in the CARSS technical scheme (25). For this study, the detection rates of 3GCRKP and CRKP reported yearly between 2014 to 2020 were collected from the CARSS database.

Meteorological data for each province was obtained from the China Statistical Yearbook (26) for the years 2014 to 2020. The data included ambient temperature, humidity, and precipitation. The data of temperature and humidity were calculated by averaging the monthly data across each year, and the precipitation was calculated by summing up 12 months across each year. To account for the seasonal temperature variation, we also calculated the average summer temperature by averaging the temperatures of June, July, and August, and the average winter temperature by averaging the temperatures of December, January, and February.

To assess the moderating effect of socioeconomic status on the relationship between temperature and AMR, we collected variables related to socioeconomic status from the China Statistical Yearbook during the same period. These variables included economic status (i.e., gross domestic product (GDP) *per capita*, consumption *per capita*, income *per capita*), healthcare resources indicators (i.e., number of hospital beds per1000 population, number of physicians and nurses per1000 population), and healthcare resources utilization indicators (i.e., hospital bed utilization rates, visit frequencies, hospitalization days, and hospitalization rates). The yearly hospital bed utilization rate was the ratio of the total bed days occupied to the total bed days available. The visit frequencies were defined as the average number of outpatient visits per person per year. The hospitalization rates were calculated as the number of inpatients divided by the number of permanent residents in the province in the same year. The yearly hospitalization days were defined as the average hospitalization days of discharged patients in the province in a year.

### 2.2. Statistical analysis

To investigate the association between ambient temperature and AMR (3GCRKP and CRKP), this study used an extended DID design (27), which can quantify the effect of ambient temperature on AMR by analyzing the concordance between differences in counts of ambient temperature and differences in AMR over time (from 2014 to 2020) at specific locations (the 31 provinces in China). A typical DID model is presented below:


$$Y_{c,t}^{A=a}=\beta_{0}+\beta_{1}a+\beta_{2}Z_{C}+\beta_{3}U_{t}+\beta_{4}W_{c,t}+\epsilon$$

where $Y_{c,t}^{A=a}$ represents the outcome in location *c* and year *t* under exposure A=a, $Z_{C}$ represents spatial confounders that vary among locations but not over the time period of the study, and $U_{t}$ represents temporal confounders that vary over time but not among locations. The $W_{c,t}$ is the confounder that fluctuates over time and among locations, and ϵ is the random error term following a normal distribution. Consequently, there will be variations in outcomes between time periods, which can be expressed as follows:


$$Y_{c,t}^{A=a}-Y_{c,t-1}^{A=a^{\prime }}=\beta_{1}\left(a-a^{\prime }\right)+\beta_{3}\;\left(U_{t}-U_{t-1}\right)+\beta_{4}\left(W_{c,t}-W_{c,t-1}\right)$$

The effects occur simultaneously in location c, which means that $\beta_{0}$ and $Z_{C}\;$ cancel out. By subtracting the discrepancies between location c and $c^{\prime }$, we obtain the following:


$$\begin{array}{l} \left[Y_{c,t}^{A=a}-Y_{c,t-1}^{A=a^{\prime }}\right]-\left[Y_{c^{\prime },t}^{A=b}-Y_{c^{\prime },t-1}^{A=b^{\prime }}\right]=\\ \;\;\;\;\;\;\;\;\;\;\beta_{1}\left[\left(a-a^{\prime }\right)-\left(b-b^{\prime }\right)\right]+\beta_{4}\left[\left(W_{c,t}-W_{c,t-1}\right)-\left(W_{c^{\prime },t}-W_{c^{\prime },t-1}\right)\right] \end{array}$$

Assuming that the variation in the rate of change of $W_{c,t}$ is uncorrelated with the variation in the rate of change of exposure in various locations, the estimate will be causal. Despite the fact that potential confounders ($W_{c,t}$) cannot be eliminated, we considered that the potential confounder variables with such characteristics could be humidity, precipitation, and their standard deviations (18). Unobserved spatial and temporal confounders were controlled by indicator variables.

In this study, the final statistical model with the DID approach is as follows:


$$\begin{array}{l} \ln\left[E\left(Y_{c,t}\right)\right]=\beta_{0}+\beta_{1}I_{c}+\beta_{2}I_{t}+\beta_{3}T_{c,t}\\ \;\;\;\;\;\;\;\;\;\;\;\;\;\;\;\;\;\;\;+\beta_{4}P_{c,t}+\beta_{5}H_{c,t}+\beta_{6}SD_{P_{c,t}}+\beta_{7}SD_{H_{c,t}} \end{array}$$

$Y_{c,t}$: the antimicrobial resistance rate in province c, year t.

$\beta_{0},\beta_{1},\beta_{2},\beta_{3},\beta_{4},\beta_{5},\beta_{6},\beta_{7}$: the intercept ($\beta_{0}$) and slopes for the linear ($\beta_{1},\beta_{2},\beta_{3},\beta_{4},\beta_{5},\beta_{6},\beta_{7}$) terms.

$I_{c}$: dummy variable for each province c.

$I_{t}$: dummy variable for each year t; we compared the year between 2014 to 2020.

$T_{c,t}$: the annual average temperature in province c, year t.

$P_{c,t},H_{c,t}$: the total annual precipitation and monthly average humidity in province c, year t, respectively; $SD_{P_{c,t}}$ and $SD_{H_{c,t}}$ are the standard deviations (SDs) of precipitation and humidity, respectively.

The DID analysis was conducted using a log-linear model. The “eliminate” option of the “gnm” package was used to specify the fixed effects terms for the provinces. The effect estimates were presented as the relative risk (*RR*) of AMR and relative 95% confidence intervals (CI) per 1°C rise in annual average ambient temperature.

Linear regression analysis was used to clarify the association between temporal trends and AMR (3GCRKP and CRKP).

To examine the potential moderating effects of other variables not included in the model on the association between temperature and AMR, we added a product term between average ambient temperature and one socioeconomic status variable into the model. We then calculated the effects based on the 10th percentile and 90th percentile of these variables. The included variables were GDP *per capita*, consumption *per capita*, income *per capita*, hospital bed utilization rates, visit frequencies, hospitalization days, hospitalization rates, number of physicians and nurses per 1,000 population, and number of hospital beds per 1,000 population.

### 2.3. Sensitivity analysis

To evaluate the reliability of the results, we conducted sensitivity analyses using a mixed model that incorporated random intercepts for province and year. We utilized the “lme4” and “lmerTest” packages for this analysis. The generalized estimating equations (GEE) were also used to analyze the association between temperature and AMR, and the autoregressive AR (1) process was used to evaluate the impact of data autocorrelation in the model. This analysis was conducted by “geepack” package. To evaluate the stability and potential delayed effect of temperature, we included current year temperature and a moving average of temperature at 1 to 2 years lag (0–1 year and 0–2 years behind) in the model. Besides, we adjusted for potential confounders by including the term $W_{c,t}$ and analyzing how various combinations of confounding variables affect the relationship between temperature and AMR. Considering the possibility of a nonlinear relationship between ambient temperature and AMR, we utilized a natural cubic spline with 4 degrees of freedom to calculate the average ambient temperature. This analysis was conducted by “spline” package.

R software (version 4.1.3) was used to perform all statistical analyses. The statistically significant level of 0.05 (two-sided) was used for all tests.

## 3. Results

### 3.1. Characteristics of antimicrobial resistance, meteorological data, and socioeconomic status

The distribution of antimicrobial resistant *K. pneumoniae*, meteorological variables, and socioeconomic status across the 31 provinces of China from 2014 to 2020 were shown in Table 1. The national average detection rate of 3GCRKP fluctuated between 26.4 to 39.1% across the 25th to 75th percentiles over the 7 years. The detection rate of CRKP across the 25th to 75th percentiles was 3.0 to 11.7%.

**Table 1:** Summary statistics for antimicrobial resistance, meteorological data, and socioeconomic status variables of 31 provinces in China from 2014 to 2020.

Variable	Mean	25th	50th	75th
Antimicrobial resistance				
3GCRKP (%)	32.9	26.4	33.0	39.1
CRKP (%)	8.1	3.0	5.8	11.7
Meteorological data				
Average temperature (°C)	14.6	10.6	15.4	17.5
Average summer temperature (°C)	25.2	23.0	26.4	27.9
Average winter temperature (°C)	2.5	−3.7	3.8	7.5
Precipitation (mm)	981.1	512.9	802.5	1408.8
Humidity (%)	65.9	57.0	64.0	76.0
Socioeconomic status				
GDP *per capita* (CNY)	60960.5	41834.0	51086.0	71101.0
Income *per capita* (CNY)	26089.0	18832.3	23102.7	28375.7
Consumption *per capita* (CNY)	18367.7	13729.6	16418.3	20463.4
Frequency of visits (per person per year)	5.6	4.3	5.2	6.1
Hospitalization days (d)	9.4	8.8	9.3	9.9
Hospitalization rate (%)	16.1	13.6	15.8	18.4
Medical staff (per thousand people)	5.3	4.5	5.2	5.9
Number of hospital beds (per thousand people)	5.7	5.0	5.7	6.3
Hospital bed utilization rate (%)	81.7	77.9	82.8	86.7

3GCRKP, third-generation cephalosporin-resistant *K. pneumoniae*; CRKP, carbapenem-resistant *K. pneumoniae*; GDP, gross domestic product; CNY, Chinese Yuan.

The annual average temperature ranged between 10.6°C to 17.5°C across the 25th to 75th percentiles. The range of annual precipitation across the 25th to 75th percentiles was 512.9 mm to 1408.8 mm, while the relative humidity ranged from 57.0 to 76.0%.

The average temperature rose by 0.3°C from 2014 to 2017 and then fluctuated from 2017 to 2020. Economic indicators such as GDP *per capita*, income *per capita*, and consumption *per capita* exhibited an upward trend between 2014 and 2017. The mean and standard deviation of annual data are shown in Supplementary Table S1.

**Figure 1:**
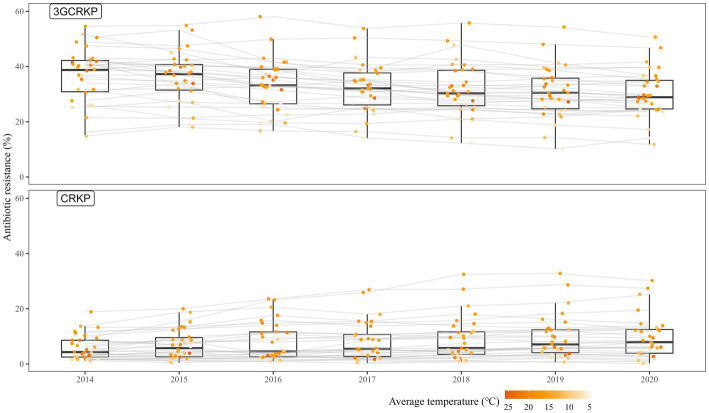
The rates of third-generation cephalosporin-resistant *K. pneumoniae*/carbapenem-resistant *K. pneumoniae* across the 31 provinces during 2014—2020. 3GCRKP, third-generation cephalosporin-resistant *K. pneumoniae*; CRKP, carbapenem-resistant *K. pneumoniae*; The gray lines present the changes in AMR in each province; the points represent the level of AMR in a specific province and year; the depth of the orange color shows the level of average temperature.

Figure 1 depicts the longitudinal fluctuations of 3GCRKP and CRKP across the 31 provinces. The prevalence of 3GCRKP was higher than that of CRKP. From 2014 to 2020, the detection rate of 3GCRKP exhibited a declining trend (regression coefficient *β* = −1.122 (95% CI:-1.739, −0.505), *p* < 0.001), whereas the detection rate of CRKP showed an upward trend (regression coefficient *β* = 0.588, (95% CI:0.135, 1.041), *p* = 0.011).

### 3.2. Association between ambient temperature and 3GCRKP/CRKP

The DID model demonstrated that the average temperature had a positive effect on both 3GCRKP and CRKP, even after controlling for precipitation and humidity (Table 2). Specifically, for every 1°C increase in the annual average ambient temperature, the rate of 3GCRKP increased by 4.7% (RR: 1.047, 95% CI: 1.031–1.082), while the rate of CRKP increased by 10.7% (RR: 1.107, 95% CI: 1.011–1.211). These results were consistent across different lag effects of average temperature (lag 0–1 and lag 0–2 years) and when considering the effects of different combinations of potential confounders (Table 2 and Supplementary Table S2). Additionally, we found that the average summer temperature was positively associated with the detection rate of 3GCRKP, but not with CRKP. However, neither the detection rate of 3GCRKP nor CRKP was significantly associated with the average winter temperature.

**Table 2:** Associations between ambient temperature and antimicrobial resistance across 31 provinces from 2014 to 2020.

Temperature variable	Strains		AMR		
			Lag 0	Lag 0–1	Lag 0–2
Average temperature	3GCRKP	RR (95% *CI*)	1.047 (1.013,1.082)	1.044 (1.006,1.083)	1.044 (1.002, 1.088)
		*p*	0.007	0.024	0.038
	CRKP	RR (95% *CI*)	1.107 (1.011,1.211)	1.218 (1.101, 1.348)	1.170 (1.047, 1.307)
		*p*	0.028	<0.001	0.006
Summer temperature	3GCRKP	RR (95% *CI*)	1.028 (1.010, 1.047)	1.026 (1.001,1.052)	1.020 (0.988,1.054)
		*p*	0.003	0.038	0.218
	CRKP	RR (95% *CI*)	1.031 (0.986,1.078)	1.095 (1.033,1.162)	1.134 (1.051,1.225)
		*p*	0.174	0.003	0.001
Winter temperature	3GCRKP	RR (95% *CI*)	1.004 (0.997,1.011)	1.008 (0.999,1.019)	1.011 (0.999,1.023)
		*p*	0.302	0.087	0.080
	CRKP	RR (95% *CI*)	1.003 (0.982,1.024)	1.024 (0.994,1.056)	1.035 (0.998,1.074)
		*p*	0.800	0.121	0.062

3GCRKP, third-generation cephalosporin-resistant *K. pneumoniae*; CRKP, carbapenem-resistant *K. pneumoniae*.

The moderating effects of precipitation and humidity on the association between the detection rates of 3GCRKP and CRKP and temperature were tested, but both the precipitation-temperature and humidity-temperature interaction terms were statistically insignificant. Specifically, the *p-*values of the interaction term between temperature and precipitation for 3GCRKP and CRKP were 0.401 and 0.684, respectively, while the *p-*values of the interaction term between temperature and humidity for 3GCRKP and CRKP were 0.066 and 0.166, respectively.

### 3.3. Moderating effects of socioeconomic variables

**Figure 2:**
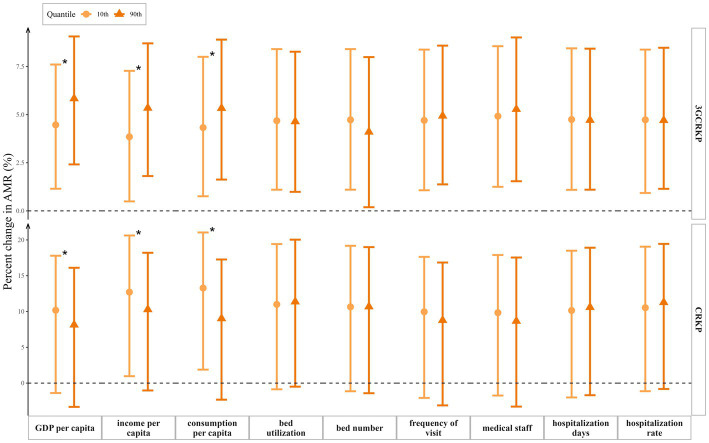
Change in the detection rates of 3GCRKP and CRKP with 95% confidence intervals per 1°C increase in average temperature at the upper and lower deciles of various socioeconomic variables: GDP *per capita* (10th percentile = 35,187.6 CNY, 90th percentile = 105,465.8 CNY), income *per capita* (10th percentile = 16,603.8 CNY, 90th percentile = 41,177.0 CNY), consumption *per capita* (10th percentile = 11,931.6 CNY, 90th percentile = 28,089.4 CNY), hospital bed utilization rate (10th percentile = 71.9%, 90th percentile = 89.9%), number of hospital beds per 1,000 population (10th percentile = 4.4, 90th percentile = 7.0), visit frequencies per person per year (10th percentile =3.9, 90th percentile =7.8), medical staff per 1,000 population (10th percentile =4.0, 90th percentile =6.5), hospitalization days (10th percentile =8.6, 90th percentile =10.5) and hospitalization rate (10th percentile =11.9%, 90th percentile =21.1%) among 31 provinces in China. ^*^ Interaction *p* < 0.05.

Figure 2 illustrated the differential effects of every 1°C increase in the average temperature on the change in the detection rates of 3GCRKP and CRKP, based on the lower decile (10th) and higher decile (90th) of socioeconomic variables. The association between the detection rate of 3GCRKP and temperature was significantly moderated by GDP *per capita* (interaction *p* < 0.001), income *per capita* (interaction *p* < 0.001), and consumption *per capita* (interaction *p* = 0.027). Similarly, the association of the detection rate of CRKP and temperature was significantly moderated by GDP *per capita* (interaction *p* = 0.002), income *per capita* (interaction *p* = 0.001), and consumption *per capita* (interaction *p* = 0.001).

However, we did not find statistically significant moderating effects of healthcare resources indicators (number of hospital beds per1000 population, number of physicians and nurses per1000 population) and healthcare resources utilization indicators (hospital bed utilization rates, visit frequencies, hospitalization days, and hospitalization rates), with *p* values all greater than 0.05.

### 3.4. Sensitivity analysis

In the mixed-effect analysis that incorporated random intercepts for province and year, we still found positive relationships between ambient temperature and the detection rate of 3GCRKP or CRKP. Specifically, we found that the detection rate of 3GCRKP increased by 2.5% (RR:1.025, 95% CI: 1.007–1.043, *p* = 0.007) and the detection rate of CRKP increased by 7.9% (RR:1.079, 95% CI: 1.019–1.143, *p* = 0.012) for every 1°C increase in annual average temperature.

When we used GEE of exchangeable correlation structure to model the association between ambient temperature and AMR, we found that both the 3GCRKP (RR:1.025, 95% CI: 1.011–1.036, *p* < 0.001) and CRKP (RR:1.053, 95% CI: 1.009–1.099, *p* = 0.017) had the positive association with ambient temperature. The positive association also had statistically significant when we used GEE of autocorrelation structure to estimate the association between ambient temperature and 3GCRKP (RR:1.022, 95% CI: 1.010–1.035, *p* = 0.001) or CRKP (RR:1.054, 95% CI: 1.013–1.098, *p* = 0.010).

Nonlinear relationships between temperature and 3GCRKP (*p* = 0.120) or CRKP (*p* = 0.501) were not detected in the non-linear models, which further confirmed the validity of our linear model (see Supplementary Figure S1).

## 4. Discussion

Our study employed DID causal inference analysis to investigate the association between temperature and AMR, and we found that higher average ambient temperature was associated with higher detection rates of 3GCRKP and CRKP. This positive association persisted across different lag effects of average temperature. Moreover, we found that socioeconomic status may moderate the association between temperature and AMR. Specifically, the effects of average ambient temperature were greater on the detection rates of 3GCRKP with a higher economic status, while were greater on the detection rates of CRKP with a lower economic status.

It showed that the detection rates of 3GCRKP and CRKP may increase in areas or during years with higher ambient temperatures in this study. Some studies had likewise supported these positive associations. A previous ecologic study showed that antibiotic resistance for *K. pneumoniae* increased by 2.2% across regions with a temperature rise of 10°C (21). Another cross-national analysis found that warmer countries with 10°C higher average minimum temperatures experienced a 0.9%/year increase in antibiotic resistance for 3GCRKP during 2000–2016 (22). Although the spatial and temporal confounding factors above studies have not been properly controlled, they do help explain the association between temperature and AMR of *K. pneumoniae*.

Even though further research is needed to clarify the mechanisms underlying the relationships between AMR and temperature, some hypotheses are proposed to explain this association. Elevated temperatures may promote the growth of bacteria, accelerating antibiotic selection and transmission through food/agriculture and environmental sources (28, 29). Furthermore, the temperature can influence resistance acquisition and transfer processes within and between hosts and environments (30). Transmission of heat-resistant genes, such as the *clpK* gene of *K. pneumoniae*, can enhance its adaptability to its surroundings (31, 32).

Our findings revealed moderating effects of economic status (GDP *per capita*, income *per capita*, consumption *per capita*) on the relationship between temperature and AMR in opposite directions. The higher socioeconomic status could strengthen the positive association between temperature and the detection rate of 3GCRKP, whereas it could weaken the positive association between temperature and the detection rate of CRKP. Higher economic status often indicates better infrastructure, governance, and more health expenditure, which can help reduce the AMR of *K. pneumoniae* (20). Furthermore, carbapenems, which are usually reserved as the “last-line agents” for anti-infection, may be used more normatively in areas with better governance (33). Although some previous studies suggested that low- and middle-income countries or regions with low GDP had a higher detection rate of 3GCRKP (34, 35), a study conducted in China did not find a significant association between GDP and 3GCRKP (36). One possible explanation of our study findings is that residents, particularly those who live in areas with higher economic levels, widely use cephalosporins due to their accessibility (37, 38), thereby facilitating AMR.

As global warming worsens, the impact of increased ambient temperature on AMR will likely become more significant (19). Dealing with high ambient temperatures as an evidenced measure should be incorporated into antimicrobial stewardship programs to slow the process of antibiotic resistance. Healthcare professionals have a crucial role in controlling global warming and reducing the incidence of AMR. Measures such as promoting the responsible use of antibiotics, reducing unnecessary healthcare (which itself has a large carbon footprint) (39–41), recycling medical materials (42), and ensuring safe sewage and medical waste treatment (43) are essential. These efforts can help mitigate the impact of global warming on AMR and contribute to combating this critical public health issue.

There are several limitations to our study. Firstly, while we controlled for spatiotemporal confounding factors by using dummy variables for each province and year, this may have increased the standard error of the impact of ambient temperature. Secondly, although we have controlled for humidity and precipitation, which are deemed as the most significant confounding factors in the association between temperature and AMR, other variables that vary over time across different locations, such as antimicrobial stewardship, were not included in our analysis. Thirdly, the modified effects of economic status on association between AMR and ambient temperature may be linked with prescription and treatment with drug; however, specific antibiotic usage data was unavailable for us. We hope that future studies could explore the effect of antibiotic consumption on the association between AMR and ambient temperature. Finally, we only used provincial population-level data with larger granularity due to the lack of individual-level data, which may have increased the random error of temperature and AMR evaluation and led to an underestimation of the association. Though the association between environmental temperature and CRKP/3GCRKP had been found by employing DID causal inference, further empirical studies especially incorporating more diverse economic regions or countries and more finely diced granularity individual data are needed to confirm their causal association.

## 5. Conclusion

This study suggested that there were positive associations between ambient temperature and detection rates of 3GCRKP and CRKP, with the associations moderated by economic status. Higher economic status strengthened the positive association between temperature and the detection rate of 3GCRKP, while weakened the association between temperature and the detection rate of CRKP. These findings highlight the need for policymakers to recognize and address the impact of high temperatures and global warming on the spread of 3GCRKP and CRKP, and to tackle the important issues of socio-economic inequalities when developing interventions and strategies for the containment of AMR.

## Data availability statement

Publicly available datasets were analyzed in this study. This data can be found here: the China Antimicrobial Resistance Surveillance System (CARSS) (http://www.carss.cn/).

## Author contributions

YZ, WL, MZ, JL, XL, LS, XY, HX, SY, and LY have substantial contributions to conception and design, funding acquisition, data collection, and interpretation of data. YZ and WL analyzed the data. YZ and LY drafted the article. MZ, JL, XL, LS, XY, HX, and SY revised it critically for important intellectual content. YZ, WL, and LY was responsible for the decision to submit the manuscript. All authors contributed to the article and approved the submitted version.

## Funding

This study was funded by grants from the China Medical Board (grant number: CMB-OC-19-337), National Natural Science Foundation of China (grant number: 72074234), Guangdong Basic and Applied Basic Research Foundation (grant number: 2022A1515011338 and 2023A1515010163), Guangzhou Basic and Applied Basic Research Program (grant number: 202201011208).

## Conflict of interest

The authors declare that the research was conducted in the absence of any commercial or financial relationships that could be construed as a potential conflict of interest.

## Publisher’s note

All claims expressed in this article are solely those of the authors and do not necessarily represent those of their affiliated organizations, or those of the publisher, the editors and the reviewers. Any product that may be evaluated in this article, or claim that may be made by its manufacturer, is not guaranteed or endorsed by the publisher.
