# Supplementary material for: The association between ambient temperature and antimicrobial resistance of Klebsiella pneumoniae in China: a difference-in-differences analysis
Source: Front Public Health. 2023 Jun 8;11:1158762. doi: 10.3389/fpubh.2023.1158762 (PMC10285064; doi:10.3389/fpubh.2023.1158762)
Supplement: Supplementary file 1 [file Data_Sheet_1.pdf]

Supplementary Material

The association between ambient temperature and antimicrobial resistance of *Klebsiella pneumoniae* in China: A difference-in-differences analysis

Yingchao Zeng† , Weibin Li† , Manzhi Zhao, Jia Li , Xu Liu, Lin Shi, Xinyi Yang, Haohai Xia, Shifang Yang, Lianping Yang*

† These authors contributed equally to this work

***Correspondence:**

Dr Lianping Yang, School of Public Health, Sun Yat-sen University, Guangzhou 510080, Guangdong Province, China. E-mail address: yanglp7@mail.sysu.edu.cn

Table S1 Temporal distribution of annual mean and standard deviation values for antimicrobial resistance, meteorological data, and socioeconomic status variables among 31 provinces in China

Variable	2014	2015	2016	2017	2018	2019	2020
**Antimicrobial resistance**							
3GCRKP (%)	36.6±10.0	35.8±9.3	33.2±9.4	32.3±8.8	31.7±9.7	30.8±9.2	30.0±9.2
CRKP (%)	6.0±4.5	7.0±5.4	7.9±6.9	8.0±7.0	8.9±7.7	9.5±7.8	9.5±7.6
**Meteorological data**							
Average temperature (°C)	14.4±5.1	14.6±5.0	14.6±5.2	14.7±5.0	14.5±5.1	14.7±5.0	14.6±5.1
Summer temperature (°C)	24.6±3.4	24.7±3.3	25.4±3.4	25.3±3.5	25.7±3.4	25.2±3.5	25.1±3.5
Winter temperature (°C)	2.0±8.2	3.2±7.9	2.4±8.2	3.1±7.8	1.4±8.4	2.6±8.0	2.9±8.2
Precipitation (mm)	913.6±568.1	1011.6±660.4	1128.9±663.2	949.5±546.0	957.6±517.4	901.3±569.3	1005.2±557.3
Humidity (%)	65.3±12.3	66.5±12.5	67.1±12.1	65.1±12.2	65.3±12.3	65.1±12.7	66.7±12.1

**Socioeconomic status**							
GDP per capita (CNY)	50742.8 ±22080.6	53083.8 ±23308.5	56766.2 ±25721.2	60855.7 ±27573.5	65253.5 ±29121.6	69235.1 ±32698.4	70786.6 ±31325.7
Income per capita (CNY)	20097.5 ±8327.8	21912.3 ±8988.5	23793.9 ±9743.6	25923.4 ±10568.9	28166.1 ±11465.4	30643.3 ±12367.0	32086.4 ±12661.0
Consumption per capita (CNY)	14640.0 ±5874.6	15856.1 ±6188.6	17206.9 ±6519.5	18371.8 ±6819.5	19926.9 ±7231.6	21560.1 ±7644.9	21012.4 ±6765.7
Frequency of visit (per person per year)	5.4±1.8	5.4±1.9	5.6±1.9	5.7±2.0	5.8±2.1	6.0±2.2	5.3±1.5
Hospitalization days (d)	9.7±0.8	9.7±0.8	9.4±0.7	9.3±0.7	9.2±0.6	9.1±0.6	9.4±0.7
Hospitalization rate (%)	14.4±3.0	14.4±3.0	15.8±3.0	16.9±3.3	17.5±3.5	18.2±3.9	15.6±3.6
Medical staff (per thousand people)	4.4±0.9	4.6±0.9	4.9±0.9	5.2±1.0	5.6±1.0	6.0±1.0	6.3±1.0
Number of beds (per thousand people)	4.9±0.6	5.1±0.6	5.4±0.7	5.7±0.8	6.0±0.8	6.2±0.9	6.5±0.9
Rate of beds utilization (%)	86.6±5.4	83.9±5.3	83.7±5.2	83.1±5.6	82.5±6.1	82.0±6.4	70.3±8.1

3GCRKP, third-generation cephalosporin-resistant *K. pneumoniae*; CRKP, carbapenem-resistant *K. pneumoniae*; GDP, gross domestic product; CNY, Chinese Yuan.

Summer (winter) temperature is an average ambient temperature during June, July and August (January, February and December) in a given year.

Model Adjustment:

$\ln[E(Y_{c,t})] = \beta_0 + \beta_1 I_c + \beta_2 I_t + \beta_3 T_{c,t} + \beta_4 P_{c,t} + \beta_5 H_{c,t}$ (A1)

$\ln[E(Y_{c,t})] = \beta_0 + \beta_1 I_c + \beta_2 I_t + \beta_3 T_{c,t} + \beta_4 P_{c,t} + \beta_5 H_{c,t} + \beta_6 GDP_{c,t}$ (A2)

$\ln[E(Y_{c,t})] = \beta_0 + \beta_1 I_c + \beta_2 I_t + \beta_3 T_{c,t} + \beta_4 P_{c,t} + \beta_5 H_{c,t} + \beta_6 SD_{P_{c,t}} + \beta_7 SD_{H_{c,t}} + \beta_8 GDP_{c,t}$(A3)

$\ln[E(Y_{c,t})] = \beta_0 + \beta_1 I_c + \beta_2 I_t + \beta_3 T_{c,t} + \beta_4 GDP_{c,t}$ (A4)

$Y_{c,t}$: the antimicrobial resistance rate in province c, year t.

$\beta_0, \beta_1, \beta_2, \beta_3, \beta_4, \beta_5, \beta_6, \beta_7, \beta_8$: the intercept and slopes for the linear terms.

I_c: dummy variable for each province c.

I_t: dummy variable for each year t; We compared the year between 2014 and 2020.

$T_{c,t}$: the annual average temperatures variable, precipitation and humidity in province c, year t.

$P_{c,t}$, $H_{c,t}$: the total annual precipitation, monthly average humidity and their standard deviation (SD) in province c, year t.
$GDP_{c,t}$: gross domestic product (GDP) per capita in province c, year t.

Table S2 Associations between temperature and antimicrobial resistance of 31 provinces from 2014 to 2020 after model adjustment.

Model adjustment	Strains		Ambient average temperature
A1	3GCRKP	RR	1.048
		(95% *CI*)	(1.014,1.083)
		p	0.006
	CRKP	RR	1.094
		(95% *CI*)	(1.003,1.195)
		p	0.044
A2	3GCRKP	RR	1.055
		(95% *CI*)	(1.022, 1.088)
		p	0.001
	CRKP	RR	1.080
		(95% *CI*)	(0.990,1.178)
		p	0.084
A3	3GCRKP	RR	1.055
		(95% *CI*)	(1.022,1.088)
		p	0.001
	CRKP	RR	1.082
		(95% *CI*)	(0.989,1.185)
		p	0.085
A4	3GCRKP	RR	1.038
		(95% *CI*)	(1.008,1.068)
		p	0.011
	CRKP	RR	1.085
		(95% *CI*)	(1.001,1.177)
		p	0.048

3GCRKP, third-generation cephalosporin-resistant *K. pneumoniae*; CRKP, carbapenem-resistant *K. pneumoniae*;

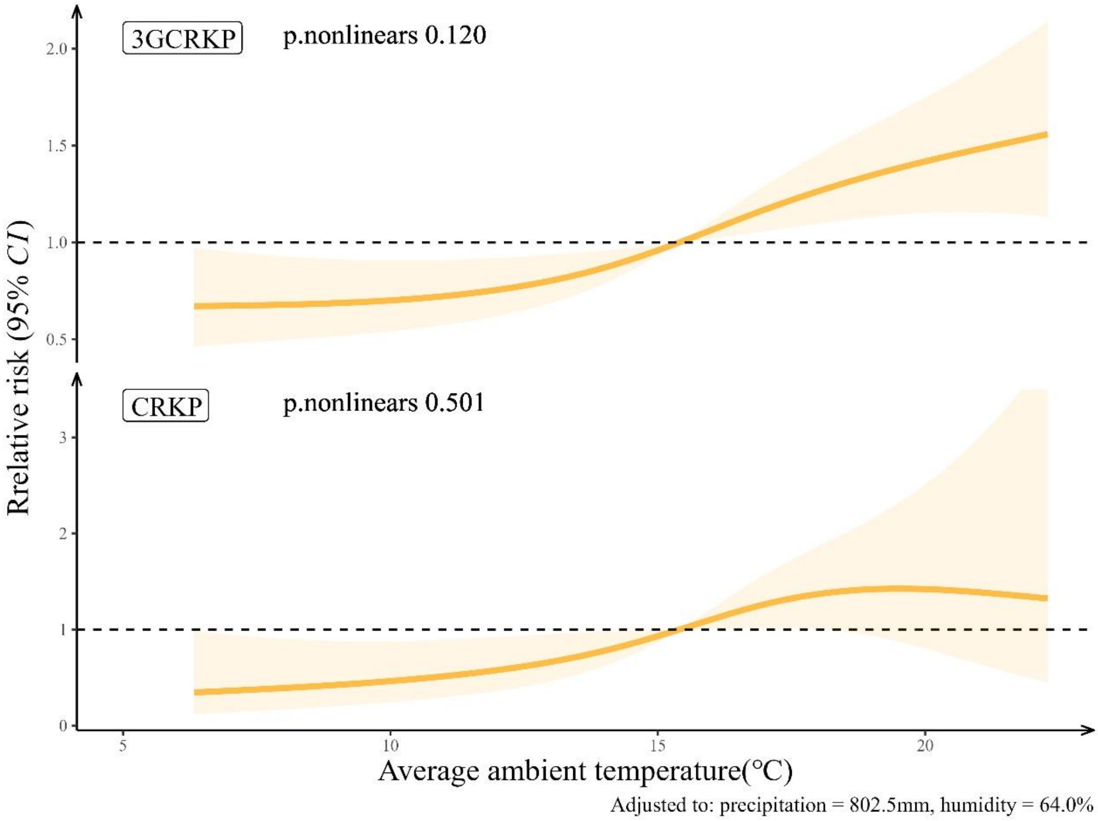

3GCRKP, third-generation cephalosporin-resistant *K. pneumoniae*; CRKP, carbapenem-resistant *K. pneumoniae.*

Figure S1 Non-linear association between average ambient temperature and third-generation cephalosporin-resistant *K. pneumoniae* / carbapenem-resistant *K. pneumoniae*
